# Hot Electrons in a Steady State: Interband vs Intraband Excitation of Plasmonic Gold

**DOI:** 10.1021/acsnano.4c03702

**Published:** 2024-07-12

**Authors:** Annika Lee, Shengxiang Wu, Ju Eun Yim, Boqin Zhao, Matthew T. Sheldon

**Affiliations:** † Department of Chemistry, Texas A&M University, College Station, Texas 77843, United States; ‡ Department of Chemistry, Emory University, Atlanta, Georgia 30322, United States; § Department of Chemistry, University of California, Irvine, California 92617, United States

**Keywords:** plasmonic, plasmons, hot electrons, steady state, interband, intraband

## Abstract

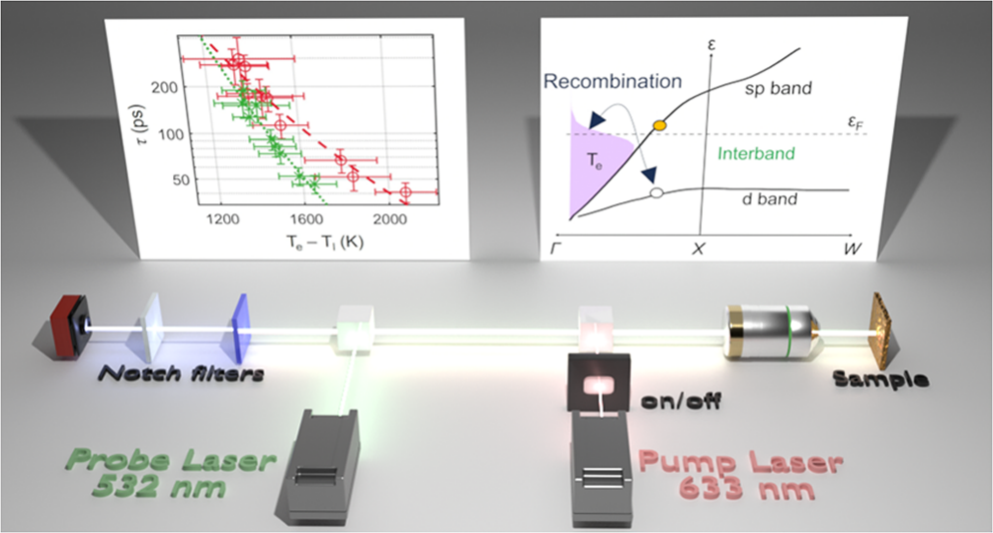

Understanding the dynamics of “hot”, highly energetic electrons resulting from nonradiative plasmon decay is crucial for optimizing applications in photocatalysis and energy conversion. This study presents an analysis of electron kinetics within plasmonic metals, focusing on the steady-state behavior during continuous-wave (CW) illumination. Using an inelastic spectroscopy technique, we quantify the temperature and lifetimes of distinct carrier populations during excitation. A significant finding is the monotonic increase in hot electron lifetime with decreases in electronic temperature. We also observe a 1.22× increase in hot electron temperature during intraband excitation compared to interband excitation and a corresponding 2.34× increase in carrier lifetime. The shorter lifetimes during interband excitation are hypothesized to result from direct recombination of nonthermal holes and hot electrons, highlighting steady-state kinetics. Our results help bridge the knowledge gap between ultrafast and steady-state spectroscopies, offering critical insights for optimizing plasmonic applications.

Metallic nanoparticles support coherent charge density waves at optical frequencies, termed plasmons, that resonantly concentrate light within nanoscale volumes.^1^ This effect has been studied considerably due to its ability to enhance light-matter interactions for photocatalysis^2−4^ and more general energy conversion processes.^5,6^ In addition to increasing optical cross sections via an antenna effect, plasmons decay nonradiatively to generate highly energetic, short-lived photocarriers that drive promising chemical reaction pathways.^7,8^ Due to their short lifetimes, ultrafast spectroscopy techniques have been crucial for revealing photocarrier generation and decay mechanisms,^9,10^ especially in the context of chemical reactions.^11−13^

Importantly, plasmon resonances are a collective electronic effect, with factors such as geometry and incident optical power significantly influencing overall behavior.^14,15^ While it is often intuitive to consider how single photons or discrete electronic transitions define the energetics of a photophysical process, in plasmonic systems, ensemble excitation and decay result in complex spatial- and time-dependent variations in electronic temperature and vibrational temperature that influence chemical behavior. Therefore, it is challenging to extrapolate from ultrafast studies that often use high instantaneous power density, if we are interested in the mechanisms underlying photochemistry observed during lower-power, continuous-wave (CW) excitation in a steady state.^16−19^ We note recent studies from Valle and co-workers that have implemented time-resolved measurements at lower incident power.^10,20,21^ Operating at lower power density is likely more relevant for practical applications, but lifetimes and decay pathways may differ greatly, contributing to the debate regarding plasmonic photochemistry.^6,22−26^ In comparison, the discrepancy between ultrafast and steady-state excitation can often be neglected for photochemistry based on the linear response of a molecular absorber or semiconductor. Yet, quantitative tools for querying plasmonic hot carrier behavior in a steady state are limited. This report aims to help draw the connection between the regime of ultrafast spectroscopy and steady-state conditions by employing an inelastic spectroscopy technique that probes the energetic distribution of electrons in the metal during CW excitation. We show how hot carrier lifetimes depend strongly on the excitation power density and, spectrally, based on interband or intraband excitation of the metal.

## Background and Theory

**Figure 1:**
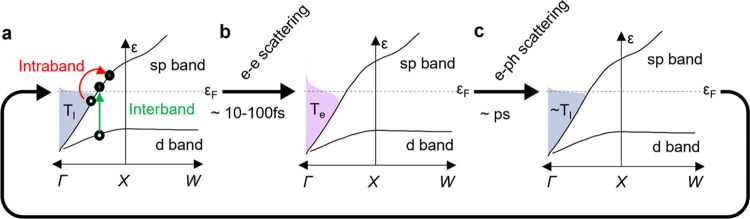
Timeline of carrier excitation and relaxation giving rise to a steady-state distribution. (a) Schematic band structure of gold with interband excitation (green, 532 nm in this study) or intraband excitation (red, 658 nm in this study) giving rise to a nonthermal distribution of electron–hole pairs. (b) These carriers thermalize into a hot electron population at temperature *T*_e_ (shaded purple). (c) Thermalized hot electrons then scatter with lattice phonons to reach a temperature *T*_l_ (shaded blue). During CW optical excitation, all of the electronic distributions depicted here are present in a steady state.

When plasmons decay, they excite short-lived, high energy electrons and holes (Figure 1) with a nonthermal energetic distribution.^7^ These carriers quickly thermalize (∼100 fs) through electron–electron (e–e) scattering,^27,28^ giving rise to a high energy, quasi-thermal population of “hot” electrons that is well approximated as a Fermi–Dirac distribution with characteristic temperatures (*T*_e_) up to a few thousand degrees.^6^ Then, within picoseconds, these hot electrons thermalize with lattice phonons (e-ph), resulting in an elevated lattice temperature (*T*_l_) in the metal.^29^

Quantifying the distinct behaviors of photocarriers during the relaxation process is crucial for understanding chemical behavior. For example, the distribution of nonthermal carriers is dictated by the band structure of the plasmonic metal.^30^ Exciting above the threshold interband transition energy (2.4 eV in gold^31^) results in the excitation of high energy but short-lived d-band holes and sp-band electrons. In contrast, lower energy intraband excitation exclusively involves transitions within the sp-band,^32,33^ so the energy distribution of nonthermal carriers is centered around the Fermi level. Unlike interband excitation, in which momentum is conserved, intraband excitation is momentum-forbidden and may require assistance from surface defects and phonons to conserve momentum.^34^ Therefore, nanoparticles with greater surface-to-volume ratio make intraband excitation more efficient compared to thin film counterparts.^30,33,35−37^

In contrast to nonthermal carriers, carriers at *T*_e_ or *T*_l_ follow Fermi–Dirac-like statistics, and the thermalization process that leads to these distributions is expected to remove any dependence on the pump wavelength besides the total amount of energy input into the system.^38,39^ Many experimental studies do not distinguish nonthermal from thermalized hot carriers, yet reactions mediated by nonthermal or thermalized carriers have different criteria in terms of excitation frequency.^40^ For instance, interband excitation accelerates hole-mediated reactions,^37^ while intraband excitation enhances electron injection over greater energy barrier heights.^35,41^ Further, the elevated lattice temperatures found in “hot spots” around nanostructures add to the confusion surrounding the chemical driving forces.^25^

Conventionally, a two-temperature model (TTM) is employed to describe the time evolution of both electronic and lattice temperatures (eq 1a,b) within plasmonic metals. The central idea is that the electron subsystem is “heated” to a high temperature from laser excitation due to its smaller electronic heat capacity, *C*_e_, compared to the lattice counterpart, *C*_l_. The excitation is then followed by electron–phonon scattering processes until the two subsystems reach the same temperature.
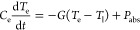
1a
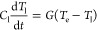
1bHere, *P*_abs_ is the absorbed optical power per unit volume, *t* is the time, and *G* is the electron–phonon interaction coefficient, which is typically determined through ultrafast studies or first-principles calculations. It should be noted that TTM assumes all electrons are thermalized among themselves at a temperature *T*_e_, and the nonthermal carriers are not included in the TTM since e–e scattering is much faster compared to e–ph scattering. One can include the kinetics of nonthermal carriers in eq 1a,b, which will lead to the extended two-temperature model (eTTM).^42,43^

Many time-resolved spectroscopy studies use the TTM to explain the time evolution of nonlinear absorption (Δ*A*/*A*) or transmission (Δ*T*/*T*), with the hot electronic temperature, *T*_e_, often reported to reach thousands of kelvins. In addition, both ultrafast and CW measurements provide unambiguous evidence for the presence of nonthermal carriers within plasmonic nanostructures.^44,45^ In contrast, the presence of thermalized hot electrons during steady-state excitation, emerging in time after nonthermal carriers are generated but before carriers are equilibrated with lattice phonons, is still debated. Whether thermalized hot electrons are present in significant numbers or at significantly elevated *T*_e_ underlies questions about the true driving forces during plasmonic photochemistry. Among a growing body of experimental signatures, our laboratory has performed both electrical measurements and spectroscopic studies, discussed more below, that provide strong evidence for a small but sustained population of hot electrons at elevated temperature *T*_e_ during CW excitation. A modified version of the TTM accounts for this small subpopulation of hot electrons, α,
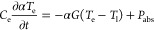
2a
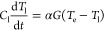
2bThe value of α ranges from 0 to 100%, with α = 100% yielding the conventional TTM in eq 1a,b. In this expression, the size of the hot electron population, α, serves to further modulate the rate of energy transfer from the hot electrons during the thermalization process. Typically, the value of α in our spectroscopic measurements during CW excitation is ∼1% or less, with the rest of electronic population equilibrated with lattice phonons. A broader implication is that all categories of electron populations, nonthermal, hot, and lattice-thermalized, are present and continuously replenished during CW illumination. Importantly, the coexistence of these distinct carrier populations may facilitate interactions among them at steady state that are relevant for hot carrier applications. This type of information can be challenging to obtain through ultrafast measurements.

## Spectral Fitting Procedure

**Figure 2:**
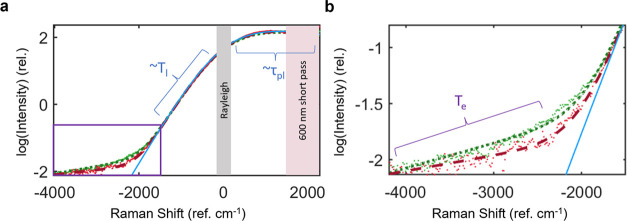
Inelastic signal from interband or intraband excitation. (a) Higher-power CW laser drove the interband (532 nm, green) or intraband (658 nm, red) transition in an Au sample, and a separate lower-power CW laser (532 nm) produced the additional inelastic counts that are plotted. The total power absorbed during either experiment was 3.26 × 10^10^ W/m^2^. The dashed lines are the fits to eq 3. The blue trace is the fit without the terms containing *T*_e_. The Rayleigh line is removed (gray), and the S signal is blocked by a 600 nm short-pass filter. (b) Zoom-in of the aS spectral region, highlighting the contribution from thermalized hot electrons.

We have developed an analytical model for interpreting the inelastic Stokes (S) and anti-Stokes (aS) signals observed from plasmonic metals during CW excitation (Figure 2). Our method provides detailed insight about plasmonic carriers in a steady state by quantifying the thermalized hot carrier temperature and population size, as well as the dephasing time of the plasmon.^7^

In recent years, many researchers have analyzed the energy distribution of the aS signal from a plasmonic metal to determine the metal temperature (Figure 2a).^46−48^ However, the accuracy of this thermometry appears to decrease at high optical power densities.^6^ In addition, there is debate about the appropriate statistical function to describe the temperature distribution: Fermi–Dirac, Bose–Einstein, or Boltzmann.^49,50^ In our experiments, we consistently observe two thermal distributions in lower and higher aS energy regimes, which may be, in part, the source of confusion (Figure 2b). By considering two separate Fermi–Dirac distributions, two distinct temperatures can be robustly fitted.^6,26^ We hypothesize that the lower energy aS signal (−500 to −2000 cm^–1^) corresponds to *T*_l_, while the higher energy aS signal is the signature of the thermalized hot electrons at *T*_e_.^6^ The overall S and aS spectrum is fit at the inelastically shifted energy, ℏω, according to the joint density of states *J*(ℏω)
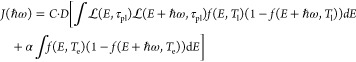
3Here, *D* is the scaling factor accounting for the collection efficiency, *f*(*E*,*T*_l_) is the Fermi–Dirac distribution of lattice-thermalized electrons at *T*_l_, *f*(*E*,*T*_e_) is the Fermi–Dirac distribution of hot electrons at *T*_e_,  is the Lorentzian function approximating the energy distribution from plasmon dephasing, with dephasing time τ_pl_. The term *C* is the photonic density of states, which can be approximated by the metal’s extinction spectrum. In practice, we obtain more accurate temperature fits by calculating *C* directly using full-wave electrodynamics simulations (finite difference time domain methods) that reproduce experimental spectra (see Supporting Information Section 1).^51^ Note that α has the same interpretation here as in eq 2a,b. A more in-depth discussion of the derivation of this fitting model and experimental validation can be found in our previous reports by Wu et al.^7,52^ The fits to experimental data using eq 3 are displayed in Figure 2. The solid blue trace is the fit without the contribution from the hot electron temperature *T*_e_, i.e., without the term scaled by α in eq 3. The brackets indicate the spectral regions with a functional form that is primarily determined by that corresponding fit parameter. It should be noted that τ_pl_ is not analyzed in this paper due to the use of a 600 nm short-pass filter, discussed below, that blocks the collection of the majority of the Stokes signal (Figure 2a).

The thermalized hot electron lifetime, τ_he_, is also indicated in the spectrum by analyzing the long-time limit of the TTM (eq 2a,b). That is, τ_he_ defines the thermalized hot electron population size, *N*, in the steady state in terms of the thermalized hot electron generation rate (eq 4). The generation rate, Γ_hot_, is experimentally defined by the absorbed incident power after accounting for a multiplicative factor corresponding to the number of thermalized hot carriers produced per absorbed photon via conservation of energy, giving
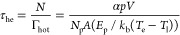
4where *N*_p_ is the number of incident photons per second, *E*_p_ is the energy provided per photon, and *k*_b_ is the Boltzmann constant. *A* is the absorptivity at the incident wavelength, *p* is the electron density, and *V* is the interaction volume. All other variables are defined in eq 2a,b and 3. A more detailed derivation of eq 4 is also provided in the Supporting Information Section 5. Because lifetime is a decisive factor governing charge transfer reactions, it is often helpful to examine trends in terms of lifetime rather than population. Note that the microscopic origin of this inelastic light signal is still a topic of debate,^53−55^ being attributed to either photoluminescence or electronic Raman scattering from the metal. Either mechanism is expected to give the same inelastic spectra.^52,56^ Please see ref (6,26,52,56−59), for a comprehensive theoretical development and several of our experiments refining this spectroscopic method.^6,26,52,56−59^

## Results

### Sample Characterization

**Figure 3:**
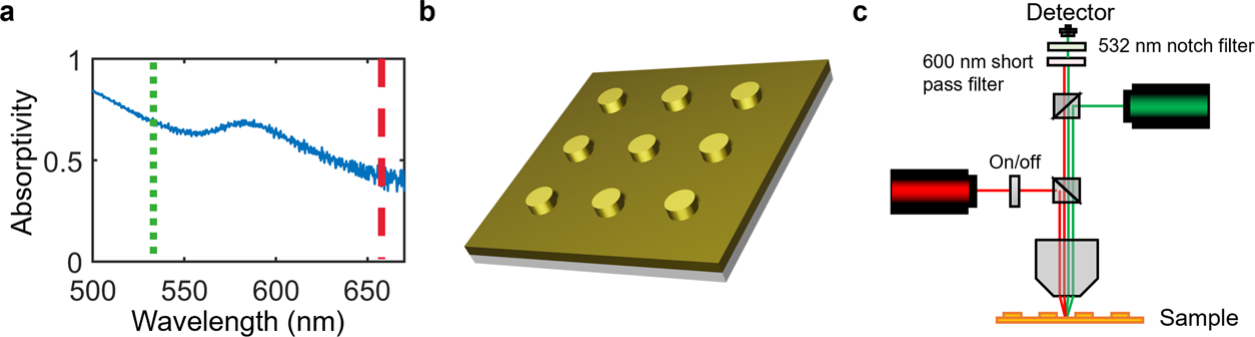
Experiment overview. (a) Absorption spectra of a nanostructure array. Samples were excited at 532 nm (green short dashed) or 658 nm (red long dashed) between 8.28 × 10^9^ and 3.69 × 10^10^ W/m^2^. (b) Schematic of nanostructure array. (c) Experiment schematic showing dual-beam CW excitation and the probe geometry.

Our study analyzes square periodic arrays of plasmonic Au nanodisks that provide high absorptivity across the visible spectrum (Figure 3a), aiding photothermal heating. Samples consist of 100 μm × 100 μm arrays of 250 nm diameter by 100 nm height disk-shaped gold nanostructures in a square lattice pattern (700 nm pitch). These were deposited on a 5 nm chromium sticking layer onto a 100 nm thick gold film on a silicon wafer using electron-beam lithography (Figure 3b) (see Supporting Information Figure S5).

### Dual-Beam Geometry

We employed a dual-beam geometry to collect inelastic spectra from the plasmonic sample (Figure 3c). For intraband excitation measurements where both 532 and 658 nm lasers are involved, the power of the probe laser is kept at 5.41 × 10^9^ W/m^2^, while for interband excitation measurements, the 532 nm laser is used as both pump and probe. We note that laser excitation at either 532 or 658 nm promotes some interband absorption and some intraband absorption. However, we estimate that at 532 nm, the interband contribution is approximately 1.47× greater than at 658 nm, motivating our simplified terminology to describe 532 and 658 nm excitation simply as interband or intraband, respectively. Our calculations estimating these respective contributions are provided in Supporting Information Section 1.3. We also attempted experiments using excitation at 405 nm to better ensure minimal contribution from the intraband transitions during interband excitation, but this proved to be incompatible with our experimental design due to significant spectral overlap between Stokes signal from the 405 nm laser and the probe spectrum (See Supporting Information Section 2).

**Figure 4:**
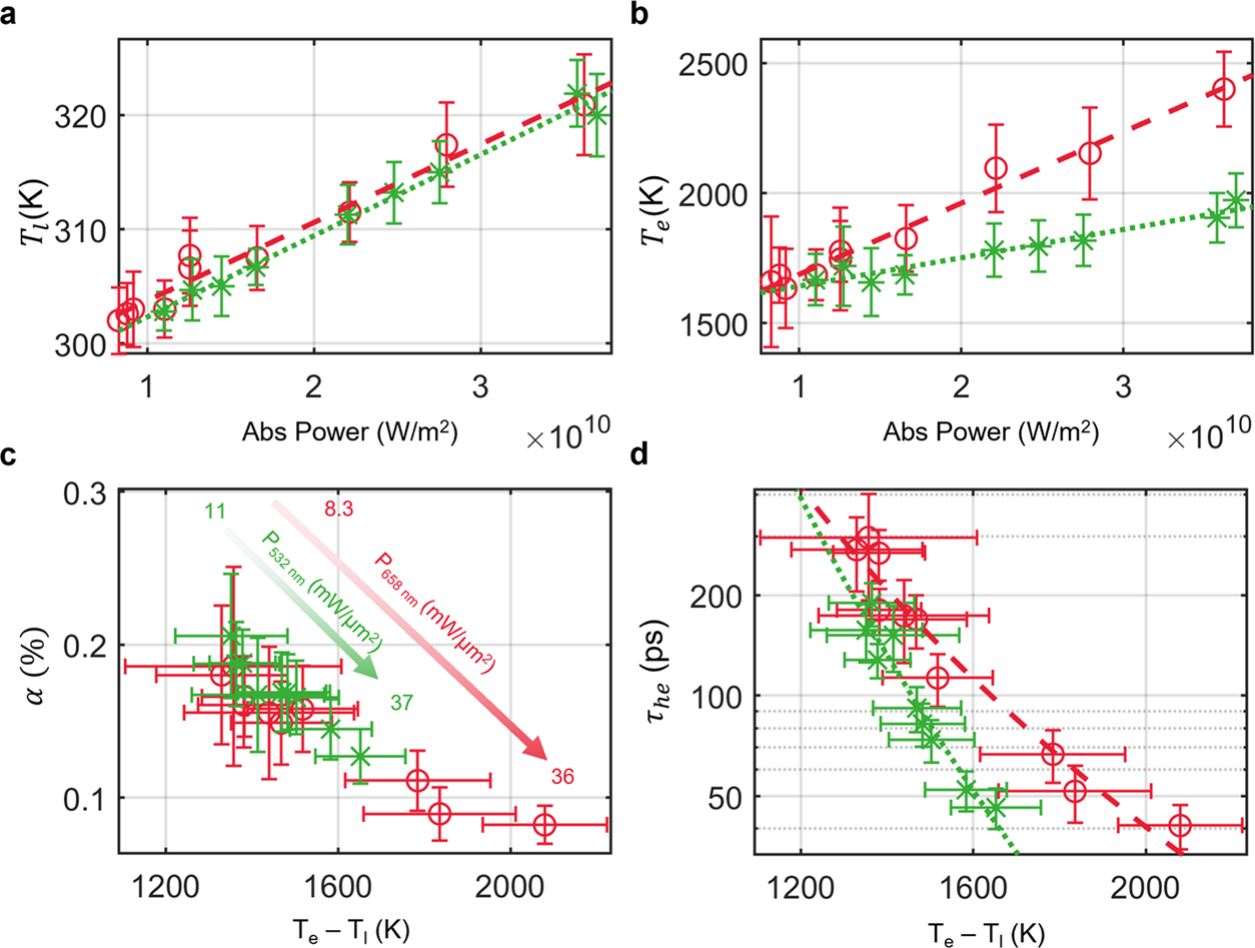
Interband data (green) and fits (green short dashed) overlaid with intraband data (red) and fits (red long dashed) with error bars of the 95% confidence interval. (a) *T*_l_ vs total absorbed power of the system. The linear fits to each temperature trend are the following: interband, *T*_l_ (K) = (7.116 × 10^–10^)*P*_abs_ (K*W^−1^m^2^) + 295.2 (K); intraband, *T*_l_ (K) = (6.85 × 10^–10^)*P*_abs_ (K*W^−1^m^2^) + 295.9 (K). (b) *T*_e_ vs total absorbed power of the system. The linear fits are used as guidelines to emphasize differences in the trends. (c) α based on the difference of *T*_e_ and *T*_l_. Respective absorbed powers are indicated on the graph. (d) τ_he_ (ps) based on the difference of *T*_e_ and *T*_l_. The exponential fits are used as guidelines to emphasize differences in the trends.

By using the same probe beam in all experiments, the fitted inelastic spectra could be collected over the same absolute wavelength range for either interband or intraband excitation, thereby eliminating factors that depended on the spectral response of the collection geometry and allowing for direct comparisons. This dual-beam geometry has been implemented successfully in previous studies,^57^ and the accuracy of the strategy is further supported by the equivalence in the fitted lattice temperatures (Figure 4a) for either excitation wavelength.

To aid reproducibility, spectra were collected at various excitation powers without any specific order of increasing or decreasing power. Additionally, a 600 nm short-pass filter was employed during both interband and intraband data collection to eliminate excess red light from the spectra. While this filter reduced saturation, it resulted in the distortion of the S side, preventing the collection of information relating to τ_pl_ (Figure 2a) (see Supporting Information Figure S6).

### Data Analysis

Representative data are displayed in Figure 2. We observe that the low energy aS spectral region associated with the lattice temperature exhibits near identical behavior up to ∼ −1800 ref cm^–1^, regardless of excitation wavelength. However, the signal at larger aS shifts exhibits differences for intraband or interband excitation that clearly exceed noise, corresponding to differences in the fitted hot electron temperature, *T*_e_. This behavior is somewhat surprising because, as discussed above, the thermalization process producing the distribution at *T*_e_ is not expected to preserve information about the pump wavelength, at least in time-resolved experiments.^38,39^ That is, the trend for either *T*_e_ or *T*_l_ is expected to depend only on absorbed power, not pump wavelength. However, the clear deviation in the higher energy aS region, specifically attributed to *T*_e_, seemingly contradicts this. These findings suggest the possibility of additional factors that may be important during steady-state excitation. To further probe this behavior, we quantify all fit parameters in eq 3 describing the thermalized electronic populations.

We observe a linear trend between *T*_l_ and absorbed power, with interband and intraband excitation resulting in *T*_l_ that overlaps within the margin of error, indicating similar behavior for the different excitation energies (Figure 4a). This is in line with the expectation that the temperature of electrons that have thermalized with the metal lattice is solely dependent on total absorbed power. Moreover, the *y*-intercept at room temperature further validates the accuracy of our measurements, affirming that *T*_l_ resolves to room temperature when there is no incident power. These trends help reinforce the accuracy of our model and our experimental approach.

For both interband and intraband excitation, *T*_e_ exhibits a monotonically increasing trend with power (Figure 4b). The values of *T*_e_ reached in our study are significant and similar in magnitude to previous studies.^6,39,46,52^ Notably, intraband excitation produces higher *T*_e_, up to 1.22× greater for the same absorbed power compared to interband excitation. Although the nonthermal carrier behavior is influenced by excitation energy as discussed above, the thermalized hot electron behavior is expected to be independent of excitation energy, mirroring the trends in *T*_l_ (Figure 4a). However, our experimental results deviate from this expectation, suggesting more complex interactions between the different electronic populations present in a steady state. An analysis of τ_he_ corroborating this interpretation is discussed further below.

The relative population, α, of carriers with temperature *T*_e_ is approximately 0.1%, which is comparable to previous studies (Figure 4c).^1,26,58^ We analyze this trend in terms of the temperature difference (*T*_e_ – *T*_l_) to help draw a comparison to the TTM (eq 1a,b). Although interband and intraband excitation produce thermalized hot electrons with similar population sizes, interband excitation requires higher powers to sustain equivalently sized steady-state populations (Figure 4c). This observation indicates that an increased generation rate of thermalized hot carriers is necessary during interband excitation to offset additional loss mechanisms present during interband pumping conditions. Furthermore, as the temperature difference (*T*_e_ – *T*_l_) increases, the thermalized hot electron population size decreases, regardless of excitation energy. Both observations can be rationalized by further analysis of the thermalized hot electron lifetime, τ_he_.

Overall, the τ_he_ observed are in the 10–100s ps range. We note that similar reported lifetimes in some time-resolved studies include signals due to the rate of cooling to the surrounding environment. The lifetimes we report here correspond to the signal from the hot electron population when the surrounding temperature in the environment is nonchanging. Therefore, this signal only provides direct information about the electron–phonon scattering rates within the metal, though it may provide indirect information about the overall dissipation of energy into other vibrational degrees of freedom.^15,60^ We elaborate below on why the time scales we measure may be longer than in ultrafast studies. In Figure 4d, the largest laser powers correspond to the greatest difference (*T*_e_ – *T*_l_) at the bottom right of the plot, and the instantaneous power density is more comparable to the pulse energy in a typical time-resolved study. Notably, for both interband and intraband excitation, τ_he_ decreases exponentially with increasing temperature difference (Figure 4d). Because (*T*_e_ – *T*_l_) is observed to increase with power, we also measure an inverse correlation between incident power and τ_he_. This trend is reflected in the decreasing α with power, and this behavior is consistently observed in previous steady-state experiments investigating thermalized hot electrons.^6,26^ This behavior aligns with the expectation according to the TTM (eq 1a,b) that, as *T*_e_ approaches *T*_l_, the rate at which the hot electrons thermalize to the phonon bath also decreases. This behavior is also analogous to justifications for excited electron lifetimes made in the context of Fermi liquid theory, which models the excitation of single electrons on the Fermi surface.^61^ More energetic electrons relax at a faster rate. However, this trend is opposite to observations in ultrafast time-resolved experiments, where a proportional relationship between incident power and τ_he_ is reported.^15,39,62^ In those ultrafast experiments, the power dependence is hypothesized to be a result of the temperature dependence of either the electronic heat capacity, *C*_e_(*T*_e_), or the electron–phonon coupling constant, *G*(*T*_e_), despite the TTM (eq 1a,b) having no explicit dependence on excitation power density.

To rationalize the discrepancy between ultrafast studies and our experiments, we analyze the time dependence of α indicated by the TTM (eq 2a,b). Our analysis is based on the fact that the temperature of the hot electrons, *T*_e_, and the lattice-thermalized electrons, *T*_l_, are not the same in a steady state during photoexcitation. We explicitly consider the temperature dependence of both *G*(*T*_e_) and the electronic heat capacity *C*_e_(*T*_e_)^43,63^ but omit the time dependence of *T*_e_, resulting in eq 5a,b. Within the framework of the TTM (eq 2a), the energy contained in the thermalized hot electron distribution is transferred to lattice phonons by a decrease of either the extensive quantity α or the intensive quantity *T*_e_. Further, both quantities α and *T*_e_ are nonchanging in a steady state. Nonetheless, by considering the time dependence of α, we gain insight into the factors that impact the relaxation time, i.e., the population decay kinetics, in the limit of nonchanging temperature. The resulting expression (eq 5a,b) can include or omit the term *P*_abs_ without impacting our interpretation, as further explained below. *P*_abs_ is omitted here for simplicity.
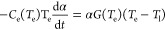
5a

After rearrangement, we obtain
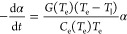
5b

The form of eq 5b shows that the decay of the thermalized hot electron population mimics the first-order kinetics in chemical reactions, that is, , and [*A*] = [*A*]_0_*e*^–*k*_1_*t*^, where [*A*]_0_ is the initial concentration of reactant. Then, the time dependence of α takes a similar form
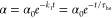
6which gives the relaxation time of thermalized hot electrons τ_he_ as
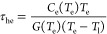
7We can see in eq 7 that temperature plays a major role in two aspects: (i) at a given lattice temperature, increasing *T*_e_ results in a decrease of the quantity ; (ii) increasing *T*_e_ results in some change to , where both *C*_e_(*T*_e_) and *G*(*T*_e_) have complex relations to *T*_e_.^43^ Using this expression, we can analyze lifetime in both CW and pulsed illumination experiments in terms of how these quantities may behave differently in the two experimental conditions. We note that including the quantity *P*_abs_ in eq 5a,b indicates that photoexcitation is a zeroth-order process, using the analogy to chemical kinetics above, without impacting our overall conclusions. A full derivation including *P*_abs_ is provided in Supporting Information Section 5 and yields an expression for τ_he_ that is more directly comparable to eq 4.

The decreasing trend of τ_he_ with increases in *T*_e_ in our steady-state experiments indicates that the power dependence of *T*_e_ in term (i) from eq 7 dominates in this regime of *T*_e_ and *T*_l_. That is, *T*_e_ increases with power, leading to a decrease in lifetime. This finding contrasts with ultrafast experiments where a monotonic increase in τ_he_ with power is observed. The use of a pulsed laser in ultrafast experiments allows for much greater *T*_e_ due to the higher peak pulse energy so that the specific dependence on the difference (*T*_e_ – *T*_l_) is negligible, with a more pronounced dependence on term (ii) instead. In principle, it may be possible to observe increasing τ_he_ under higher laser powers during CW excitation, like in ultrafast studies, though in practice, our samples degrade at higher power density due to melting.

In addition, our data reveals that as *T*_e_ increases, there is a growing deviation in τ_he_ based on interband or intraband excitation (Figure 4d). At the same temperature difference (*T*_e_ – *T*_l_) of 1652 K, intraband excitation results in hot electron lifetimes that are 2.34× longer than during interband excitation. While intraband excitation produces nonthermal holes and nonthermal electrons symmetrically around the Fermi energy, interband excitation leads to the preferential generation of higher-energy nonthermal holes (Figure 1). We hypothesize that the more rapid decay of the thermalized hot electrons during interband excitation is due to direct recombination of hot electrons with the larger population of more reactive nonthermal holes. The result is shorter lifetimes τ_*he*_ and diminished energy contained within the thermalized hot electron distribution, leading to cooler hot electron temperatures for the same incident power. In comparison, this effect may not be observed during ultrafast measurements. The nonthermal population is already decayed by the time the thermalized hot electron population has emerged, making direct recombination between them unlikely to be observed in the relatively longer-time decay signal of the hot electrons. However, quantitative analysis of differences in the efficiency of the conversion on nonthermal carries into hot electrons at very early times in time-resolved studies may be useful for further elucidating the mechanism we propose here. Further, these studies can distinguish it from other nonlinear processes that may be important, such as Auger scattering, which may have efficiency that depends on intraband or interband excitation. The lattice-thermalized electron temperature, *T*_l_, remains unaffected by differences in the electronic energy distribution in a steady state because *T*_l_ is determined by the much slower process of thermally conducting the total input power into the surrounding environment.

## Conclusions

Our investigation has provided insight into the kinetics of different electron populations in plasmonic metals under continuous-wave (CW) illumination. Our analysis is based on a spectral fitting procedure that quantifies different energetic distributions that give rise to the inelastic light signal coming from a plasmonic metal. The interplay between nonthermal and thermalized hot electrons, alongside lattice-thermalized electrons, is found to significantly impact the energy distribution and carrier lifetimes in a steady state during photoexcitation. A major insight is the monotonic increase in hot electron lifetime as the hot electron temperature is decreased. We rationalize this behavior as a manifestation of the inverse relationship between electronic lifetime and hot electron temperature that is predicted in the TTM. Further, we have probed the inelastic signal that is produced during interband or intraband excitation. We find a significant increase of 1.22× in the hot electron temperature during intraband excitation at the same power density compared with interband excitation, as well as a 2.34× increase in the hot carrier lifetime. We hypothesize that the shorter lifetime during interband excitation is due to the direct recombination of photoexcited nonthermal holes and thermalized hot electrons. Because these carrier populations are produced at distinct times after photoexcitation, this interaction may be indicative of dynamic processes that differ from ultrafast conditions. We believe our findings may hold significance in the context of hot electron-mediated reactions, as longer lifetimes and greater temperatures facilitate more efficient electron transfer.

## Methods

### Nanostructure Fabrication

Prior to fabrication, a silicon substrate was cleaned using base piranha. A 5 nm chrome layer and then a 100 nm gold layer were evaporated (Lesker PVD electron-beam evaporator) onto the silicon substrate. Next, 950 poly(methyl methacrylate) (PMMA) A4 was spin-coated onto the substrate as the electron-beam resist layer. Electron-beam lithography (TESCAN MIRA3 EBL) was performed to pattern the 100 μm × 100 μm nanodisk array into the resist. After development, a 5 nm chrome adhesion layer was deposited on the surface of the exposed PMMA, followed by a 100 nm layer of gold. Finally, liftoff was performed in acetone using pipet pumping, leaving only the nanodisk array on the surface of the substrate.

### Raman Spectroscopy Configuration

Raman spectra were taken using a confocal microscope system (Witec RA300) and spectrometer (UHTS300, grating = 300 g/mm). A schematic of the setup is featured in Figure 3c of the main article. A holographic 532 nm notch filter (RayShield Coupler, Witec) was used to reduce Rayleigh scattering, and a short-pass 600 nm filter (Sputtered Edgepass Filter, Thorlabs) was used to prevent saturation. For intraband measurements, the probe source was a 532 nm CW Nd:YAG laser coupled through a fiber coupler, and the excitation source was a 658 nm CW semiconductor laser coupled through free space. The 532 nm laser had a lower intensity than the 658 nm laser. Both beams were focused using either a 100× objective (Zeiss EC Epiplan Neofluar, NA = 0.9, WD = 0.31 mm) or a 50× objective (Zeiss EC Epiplan). The beam diameter for the 532 nm laser is approximately 400 nm under the 100× objective and 620 nm under the 50× objective. The beam diameter for the 658 nm laser is approximately 450 nm for the 100× objective and approximately 2240 nm for the 50× objective. The beam diameter was determined at 1/*e*^2^ intensity. Inconsistencies in 658 nm spot size are due to changes in the free space path between sets of measurements. Lasers were aligned and focused by maximizing the aS signal and correlating lattice temperatures for maximum heating.

Interband measurements were taken utilizing the 532 nm laser only at power densities between 1.01 × 10^10^ and 3.69 × 10^10^ W/m^2^ and then subtracting out the dark spectra. Intraband measurements were taken utilizing the 658 nm laser at power densities between 8.28 × 10^9^ and 3.62 × 10^10^ W/m^2^ at high power and the 532 nm laser at 5.41 × 10^9^ W/m^2^ power simultaneously. This method is adapted from our previous study.^57^ A spectrum of only the 658 nm laser is then taken, and the 658 nm contribution is then subtracted out, leaving only the signal collected by the 532 nm laser. This was done to ensure consistency in our probe excitation. All data collection was conducted under atmospheric conditions and optimized at hot spots between nanostructures.

## Supporting Information Available

Details of our LDOS calculations; further interband excitation attempts; sample analysis; estimated plasmon dephasing times; and detailed derivations are provided (PDF)

## Abbreviations

TTM: two-temperature model
S: stokes
aS: anti-Stokes
CW: continuous wave
