# Supplementary material for: Hot Electrons in a Steady State: Interband vs Intraband Excitation of Plasmonic Gold
Source: ACS Nano. 2024 Jul 12;18(29):19077–85. doi: 10.1021/acsnano.4c03702 (PMC11271177; doi:10.1021/acsnano.4c03702)
Supplement: Supplementary file 1 — nn4c03702_si_001.pdf [file nn4c03702_si_001.pdf]

Supporting Information for

Hot Electrons in a Steady State: Interband vs. Intraband Excitation of Plasmonic Gold

Annika Lee[1], Shengxiang Wu[2], Ju Eun Yim[1], Boqin Zhao[1], Matthew T. Sheldon[1,3]*

[1]Department of Chemistry, Texas A&M University, College Station, TX 77843, USA

[2]Department of Chemistry, Emory University, Atlanta, GA 30322, USA

[3]Department of Chemistry, University of California, Irvine, CA 92617, USA

[*]Corresponding author(s). E-mail: m.sheldon@uci.edu

1. Full wave electromagnetic simulations

1.1 Simulations Setup

Three-dimensional full wave electromagnetic simulations were performed using finite difference time domain (FDTD) methods (Lumerical, Ansys Inc.). A 100 nm height and 100 nm radius Au nanodisk on 100 nm thick Au film defined the simulation geometry. This differs slightly from actual geometry to obtain the most accurate simulation to experimental spectra with differences resulting from changes in refraction index. We ignored the 5 nm Cr sticking layer because it has a negligible effect on the overall optical properties of the system and requires finer mesh sizes. Periodic boundary conditions were applied on all sides perpendicular to the substrate with a simulation region of 700 nm x 700 nm, representing the periodicity of the nanostructure. The substrate was on x-y plane. A plane wave source was injected from above, along z direction, normal to the substrate. 2-D z-normal monitors were placed above the source plane for collecting backscattered signal and below the Au film for transmitted signal

from the nanostructure. 3-D monitors were set up to collect the electric field data on Au. The permittivity values used were from the work of Johnson and Christy.[1]

1.2 Total absorption spectrum

First, we calculated the simulated total percent absorption spectrum and compared it with the experimental absorption spectrum. This process helped confirm the validity of the further calculation of the LDOS enhancement of the nanostructure. The absorption spectrum was calculated by taking one minus the overall backscattered percentage of power and transmitted percentage of power. The transmitted percentage was negligible due to the opacity of the nanostructure. The result is shown in Figure S1. The percent absorption was plotted in the scale of 0 to 1. Although the simulated intraband resonance is slightly red-shifted and the absorption intensity is smaller than that of the experimental spectra, the general shape is very similar. We believe that the simulated geometry would give good estimates of LDOS enhancement over the spectral region of interest.

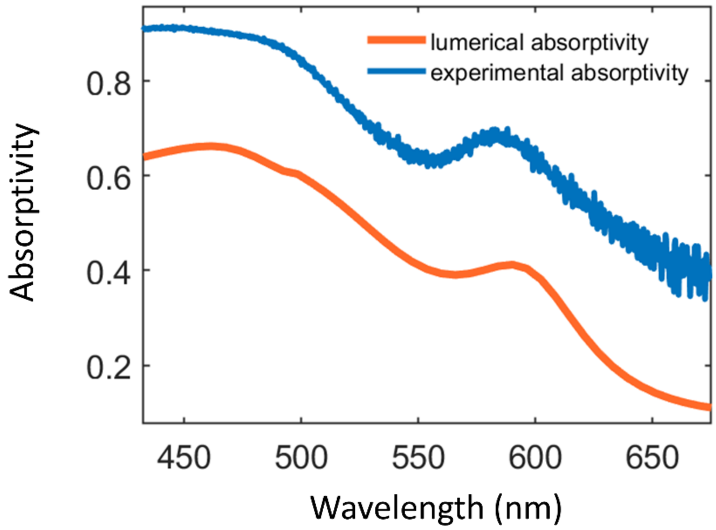

Figure S1. Absorption spectrum taken from the substrate (blue) overlayed with simulated absorption spectrum (orange).

1.3 Calculating Interband vs. Intraband Contributions

We isolated the intraband contribution to the complex dielectric of Au metal, ϵ_{rf}, using the Drude model, employing the values provided by Rakić *et al.*.[2]

$$\epsilon_{rf}(\omega) = 1 - \frac{{\Omega_p}^2}{\omega(\omega - i\Gamma_0)} \tag{S1}$$

Here ${\Omega_p}^2$ is the plasma frequency associated with intraband transitions, ω is angular frequency, and Γ_0 is the intraband damping constant. We use this to calculate real (n) and imaginary (k) parts of the refractive index.

$$n = \frac{1}{\sqrt{2}}\left[({\epsilon_{r1}}^2 + {\epsilon_{r2}}^2)^{1/2} + \epsilon_{r1}\right]^{1/2} \tag{S2a}$$

$$k = \frac{1}{\sqrt{2}}\left[({\epsilon_{r1}}^2 + {\epsilon_{r2}}^2)^{1/2} + \epsilon_{r1}\right]^{1/2} \tag{S2b}$$

Based on this simplified refractive index, we numerically determined the absorption spectra of the experimental geometry as illustrated in section 1.2 above. The comparison of the absorption using the simplified refractive index (Drude contribution only) against the full numerical simulation described in section 1.2 above, allows us to estimate the relative contribution from interband and intraband absorption at the two pump wavelengths in the experiment (see figure S2).

By this estimate, at 532 nm the interband contribution to absorption is approximately 1.47x greater than at 658 nm. We note that our calculation method may overestimate the interband contribution at 658 nm, as this transition is expected become inactive at wavelengths longer than 680 nm.[3, 4] For the sake of simplicity, in the manuscript we describe the 532 nm excitation as the interband excitation and the 658 nm excitation as the intraband excitation.

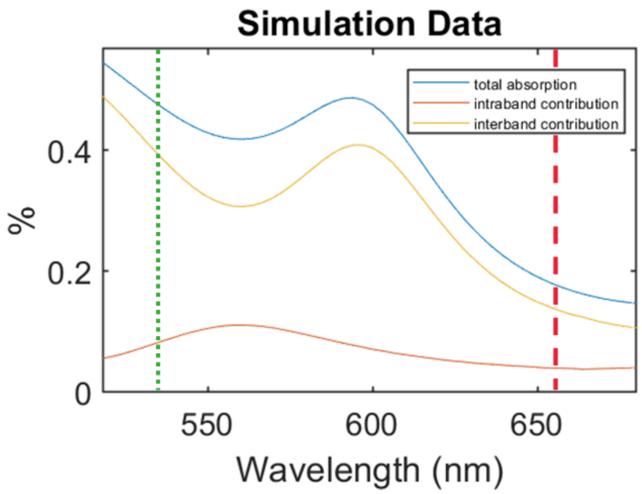

Figure S2. Interband vs Intraband contribution to absorption**.** Calculated absorption spectra using full-wave electromagnetic simulations (FDTD, Lumerical). The interband contribution (yellow) is calculated by subtracting the intraband contribution (red) from the total absorption (blue). The experimental pump wavelengths at 532 nm (green) and 658 nm (red) are depicted with vertical lines.

1.4 LDOS enhancement spectrum

The LDOS enhancement is also called the Purcell factor, which is the enhancement of the power emitted by a dipole into an inhomogeneous optical environment over that into the vacuum. We used the Lorentz reciprocity relation to calculate LDOS enhancement because the Raman signal can be described as the emission of electronic excitation of Au nanostructures into a lossy environment by means of propagating modes. The detailed derivation on this formalism was described elsewhere.[5]

The Purcell factor $F_k(r_0)$ for the emission into mode k is defined by

$$F_k(r_0) = \frac{P(r_0)_{k,l}}{P_0} = \frac{3\pi c}{4\mu_0\omega^2}\frac{\left|E(r_0)_{-k,l}\right|^2}{P_{-k}}, \tag{S3}$$

where $P(r_0)_{k,l}$ is the power emitted by an l-polarized imaginary dipole source at a position of r_0 propagated to mode k, and P_0 is the power emitted by the same dipole source to the vacuum. c is the

speed of light, μ_0 is the vacuum permeability, ω is the angular frequency, $E(r_0)_{-k,l}$ is l-component the local electric field evaluated at the position of r_0 generated by the reciprocal incident plane wave of mode k (denoted as $-k$ including the propagation direction into the system). Finally, P_{-k} is the power carried by that incident plane wave.

If we consider the incoherent Raman signal as the power emitted by an unpolarized imaginary dipole source as in the case of thermal excitation, then just the powers emitted by randomly oriented dipoles can be added separately. In other words, we can obtain the unpolarized fields by sum the results incoherently.

$$F_k(r_0) = \frac{P(r_0)_{k,x} + P(r_0)_{k,y} + P(r_0)_{k,z}}{3P_0} = \frac{\pi c}{4\mu_0\omega^2}\frac{\left|E(r_0)_{-k,x}\right|^2 + \left|E(r_0)_{-k,y}\right|^2 + \left|E(r_0)_{-k,z}\right|^2}{P_{-k}}. \tag{S4}$$

The results of $F_k(r_0)$ at each point in space excluding the Au structures are then sum and averaged over the entire simulation region by taking the volume integral. By evaluating the average Purcell factor for different excitation wavelength of interest, the LDOS enhancement ($\frac{\rho}{\rho_0}$) spectra is obtained. The blue plot in Figure S3 is normalized $\frac{\rho}{\rho_0}$ to its maximum in the spectral region of interest.

$$\frac{\rho}{\rho_0} = \int_V F_k(r)\, dV. \tag{S5}$$

The final LDOS enhancement plot used in the main text is shown with the red plot. We noticed that the blue plot calculated directly from the simulated geometry gave an incorrect lattice temperature. We corrected the unreasonably high LDOS intensity at the resonance by artificially decreasing the overall intensity at around 606 nm. We believe this reflects on the imperfections across the nanostructures that

cannot be represented in simulations, and the adjusted one better represents the physical picture of the optical environment of the fabricated nanostructures and this is reflected in the accuracy of the lattice temperatures given (Figure 4a in the main article).

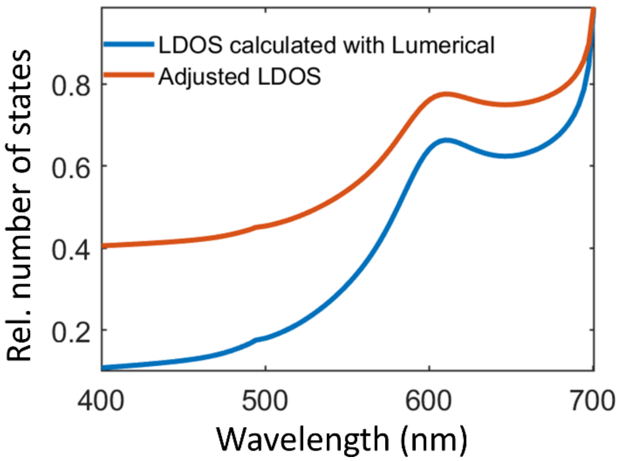

Figure S3. LDOS enhancement spectra calculated in the simulated geometry (blue) overlaid with the final adjusted LDOS enhancement for the substrate (orange). Total number of states is not required for this calculation and therefore normalized for comparison.

2. Attempts at Reducing Intraband Contribution during Interband Excitation

Significant attempts were made to further distinguish signals due to interband or intraband excitation, achieved in principle, by using a more blue-shifted excitation wavelength compared to the 532 nm probe laser in this study. However, when exciting plasmonic metals there is a broad SERS Stokes signal. This SERS Stokes signal is examined in more detail in a previous manuscript from our laboratory (*J. Appl. Phys.* **2021,** *129* (17) 173103),[6] and it is primarily due to an inelastic signal from the non-thermal electronic population. During a blue-shifted pump, this signal overlaps with the thermalized hot electron signal contained in the 532 nm probe spectrum, but it is far stronger. Ideally, using our experimental methods the undesirable Stokes signal from the blue laser could be subtracted out, but because it is extremely sensitive to small changes in focus and temperature, even small fluctuations overwhelm the

thermalized hot electron signal produced from the 532nm probe laser. Nonetheless, we attempted the same experiment using a 405 nm laser in hopes of reducing the overlapping signals (see below). However, when the pump wavelength was moved closer to the UV, the SERS signal became even stronger. When exciting at 405 nm this SERS signal still extended to –2000 cm^{-1} within the probe spectrum, even at the lowest pump power for our study. In our paper we fit the thermalized hot electron signal up to –4000 cm^{-1}. Additionally, our microscope is not equipped to handle UV excitation at even higher energy. Representative spectra showing the problematic spectral overlap are displayed below.

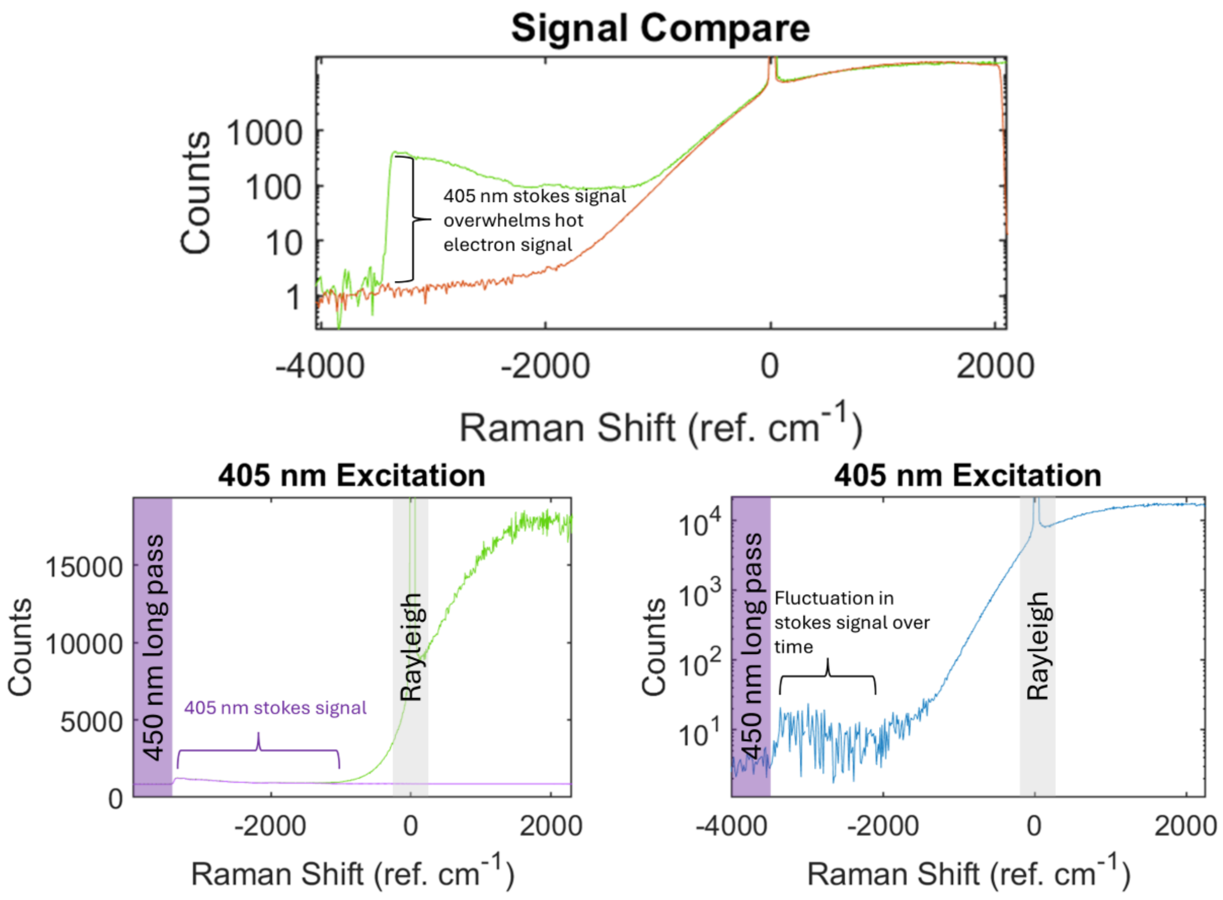

Figure S4. The 405 nm SERS signal overwhelms the anti-Stokes signal of the 532 nm probe laser. TOP: The 405 nm Stokes SERS signal with the probe spectrum (green) shows the contribution of the SERS

signal that is at least 100x greater than the thermalized hot electron signal produced by only 532 nm excitation (orange). BOTTOM: By subtracting the spectrum with only 405 nm excitation (purple) from the signal with the 405 nm pump excitation and the 532 nm probe excitation (green) we replicate the procedures used to analyze trends at other pump wavelengths in our manuscript (blue). However, small fluctuations in the 405 nm SERS signal are still greater than anti-Stokes signal required for our analysis.

3. Sample Characterization

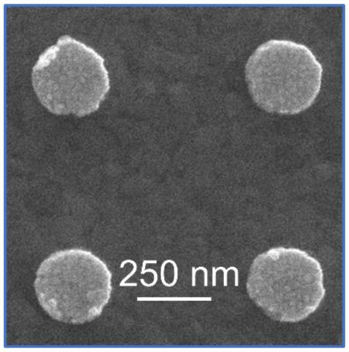

Figure S5. Scanning Electron Microscopy was used to characterize the sample to ensure accurate shape and dimensions.

4. Estimated Plasmon dephasing time

Precise dephasing times cannot be obtained unlike in previous experiments due to the 600 nm short-pass filter and red laser saturation. However, the slight differences in plasmon dephasing time due to excitation energy has a very minor effect on the other obtained values, and therefore is not important in the context of this experiment. Visually, the intraband data seems to have a slightly greater plasmon dephasing time (Figure S6), though this is difficult to confirm without access to higher Stokes shifts. Full fits of the stokes data can be seen in previous works.[6]

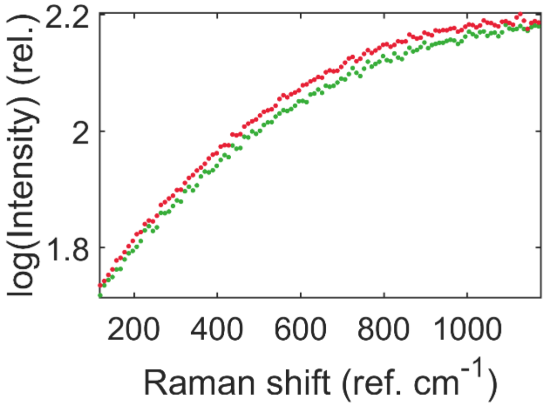

Figure S6. Raw Stokes side Raman signal. Plasmon dephasing time is estimated to be roughly 18 fs for both interband (green) and intraband (red) data.

5. Model development

Here we provide the complete model development in detail regarding the hot electron lifetime in the main text.

The seed of our argument starts with the well-accepted two-temperature model (TTM):

$$C_e \frac{dT_e}{dt} = G(T_e - T_l) + P_{abs} \tag{S6a}$$

$$C_l \frac{dT_l}{dt} = G(T_e - T_l) \tag{S6b}$$

Where P_{abs} is the absorbed optical power per unit volume, t is time, and G is the electron-phonon (e-ph) interaction coefficient. The form is equation S6 is widely used, however, one should note that both heat capacities (C_e, C_l) and e-ph interaction coefficient G is a function of temperature.

As we elaborated in the main text that a small yet sustained population of hot electrons at T_e is present at steady state while the rest of the electrons are equilibrated with lattice phonons at T_l (the

even smaller population of non-thermal carriers). We termed this small population α and it is then inserted into equation S6 which yields the steady state version of TTM:

$$C_e \frac{d\alpha T_e}{dt} = \alpha G(T_e - T_l) + P_{abs} = 0 \tag{S7}$$

When α = 100%, equation S7 gives the original TTM model S6a. In our measurements, the value of α during CW excitation is ~1% or less. The right-hand side of equation S7 contains one decay channel and the excitation of hot electrons. It is noted that when in transient measurements, the excitation term P_{abs} only lasts within a pulse period, and it is the decay channel – the electron-phonon scattering, that gives the rich time-dependent features in ultrafast spectra. So if we follow the similar manner and isolate the time-dependence of α as we justified in the main text, equation S7 can be recast into

$$C_e(T_e) \frac{d\alpha T_e}{dt} = -\alpha G(T_e)(T_e - T_l) \tag{S8}$$

Also note that the temperature-dependence of C_e and G are added. Equation S8 suggests an exponential decay of hot carrier population which is indeed observed in many ultrafast studies. Without making any assumptions about the different timescales, rearrange equation S8 yields

$$C_e(T_e)\left(\frac{\partial \alpha}{\partial t} T_e + \frac{\partial T_e}{\partial t} \alpha\right) = -\alpha G(T_e)(T_e - T_l) \tag{S9a}$$

Simple rearrangement of equation S9a yields

$$-\frac{\partial \alpha}{\partial t} = \left[\frac{G(T_e)(T_e - T_l)}{C_e(T_e)T_e} + \frac{1}{T_e}\frac{\partial T_e}{\partial t}\right]\alpha \tag{S9b}$$

The second term $\frac{1}{T_e}\frac{\partial T_e}{\partial t}$ is far smaller than the first term $\frac{G(T_e)(T_e - T_l)}{C_e(T_e)T_e}$ as steady-state conditions entail that $\frac{\partial T_e}{\partial t}$ is close to zero. With only the first term included, we obtain the following expression

$$-\frac{\partial \alpha}{\partial t} = \frac{G(T_e)(T_e - T_l)}{C_e(T_e)T_e} \tag{S10}$$

We make analogies with chemical kinetics in the main text by realizing that equation S10 is similar to a first-order chemical kinetics, that is, $-\frac{d[A]}{dt} = k_1[A]$, and $[A] = [A]_0 e^{-k_1 t}$. Then, the time dependence of α takes a similar form:

$$\alpha = \alpha_0 e^{-k_1 t} = \alpha_0 e^{-t/\tau_{he}} \tag{S11}$$

which gives the relaxation time of thermalized hot electrons τ_{he} as

$$\tau_{he} = \frac{C_e(T_e)T_e}{G(T_e)(T_e - T_l)} \tag{S12}$$

Temperatures are present in both numerator and denominator in equation S12 and thus may give contradict trends of hot electron lifetime in ultrafast and CW measurements since the temperature reached in these two distinct scenarios is dramatically different.

We also note that we have employed the conservation of energy argument in the main text (equation 4) to empirically extract the lifetime of hot electrons. We now show that such argument is equivalent to the same chemical kinetics analogy if we do not drop the excitation term in equation S7.

$$-C_e(T_e)\frac{d\alpha T_e}{dt} = \alpha G(T_e)(T_e - T_l) - P_{abs} \tag{S13}$$

Similarly isolate the time-dependence of α and keep the left-hand side only contains $-\frac{d\alpha}{dt}$

$$-\frac{d\alpha}{dt} = \frac{G(T_e)(T_e - T_l)}{C_e(T_e)T_e}\alpha - \frac{P_{abs}}{C_e(T_e)T_e} \tag{S14}$$

Therefore, the kinetics of hot carriers is simply a combination of a first-order reaction (electron-phonon scattering) and a zeroth-order reaction (excitation). Since for the zeroth-order reactions, the kinetics is $-\frac{d[A]}{dt} = k_2[A] \Leftrightarrow [A] = [A]_0 - k_2 t$, the overall kinetics of the hot carriers, based on equation S14, is

$$\alpha = \alpha_0 e^{-k_1 t} + \frac{P_{abs}}{C_e(T_e)T_e} t \quad \text{(S15)}$$

where α_0 is the steady-state population of hot electrons, we set the whole equation in S15 equals to α_0 since it is at steady state, which will lead to

$$\alpha_0 e^{-k_1 t} + \frac{P_{abs}}{C_e(T_e)T_e} t = \alpha_0 \quad \text{(S16)}$$

Furthermore, if we Taylor expand the first order term, the equation S16 is approximated (within first-order) as

$$\alpha_0 e^{-k_1 t} + \frac{P_{abs}}{C_e(T_e)T_e} t \approx \alpha_0(1 - k_1 t) + \frac{P_{abs}}{C_e(T_e)T_e} t = \alpha_0 \quad \text{(S17)}$$

Since rate constant k_1 and relaxation time τ_{he} are inversely proportional, $k_1 = \frac{1}{\tau_{he}}$, we can get τ_{he} as

$$\tau_{he} = \frac{\alpha_0}{\frac{P_{abs}}{C_e(T_e)T_e}} = \frac{\alpha_0 pV}{N_p A E_{photon} / C_e(T_e)T_e} \quad \text{(S18)}$$

Which is exactly (within first-order approximation $C_e \propto k_b$) the empirical equation we used in the main text.

(1) Johnson, P. B.; Christy, R. W. Optical Constants of the Noble Metals. *Physical Review B* **1972**, *6* (12), 4370-4379. DOI: 10.1103/PhysRevB.6.4370.
(2) Rakić, A. D.; Djurišić, A. B.; Elazar, J. M.; Majewski, M. L. Optical properties of metallic films for vertical-cavity optoelectronic devices. *Appl. Opt.* **1998**, *37* (22), 5271-5283. DOI: 10.1364/AO.37.005271.
(3) Santiago, E. Y.; Besteiro, L. V.; Kong, X.-T.; Correa-Duarte, M. A.; Wang, Z.; Govorov, A. O. Efficiency of Hot-Electron Generation in Plasmonic Nanocrystals with Complex Shapes: Surface-Induced Scattering, Hot Spots, and Interband Transitions. *ACS Photonics* **2020**, *7* (10), 2807-2824. DOI: 10.1021/acsphotonics.0c01065.
(4) Kolwas, K.; Derkachova, A. Impact of the Interband Transitions in Gold and Silver on the Dynamics of Propagating and Localized Surface Plasmons. *Nanomaterials* **2020**, *10* (7), 1411.
(5) Schulz, K. M.; Jalas, D.; Petrov, A. Y.; Eich, M. Reciprocity approach for calculating the Purcell effect for emission into an open optical system. *Opt Express* **2018**, *26* (15), 19247-19258. DOI: 10.1364/oe.26.019247 From NLM.
(6) Wu, S.; Cheng, O. H.-C.; Zhao, B.; Hogan, N.; Lee, A.; Son, D. H.; Sheldon, M. The connection between plasmon decay dynamics and the surface enhanced Raman spectroscopy background: Inelastic scattering from non-thermal and hot carriers. *Journal of Applied Physics* **2021**, *129* (17), 173103. DOI: 10.1063/5.0032763 (acccessed 2022/08/14).
